# PS II Subunit P in *Lilium pumilum* (LpPsbP) Confers Saline-Alkali Resistance to the Plant by Scavenging ROS

**DOI:** 10.3390/ijms24043311

**Published:** 2023-02-07

**Authors:** Yibo Jing, Yu Song, Shangwei Ji, Ling Zhang, Zongying Wang, Yi Dong, Yang Xu, Shumei Jin

**Affiliations:** 1 Key Laboratory of Saline-Alkali Vegetation Ecology Restoration, Ministry of Education, College of Life Sciences, Northeast Forestry University, Harbin 150000, China; 2 Aulin College, Northeast Forestry University, Harbin 150000, China

**Keywords:** *Lilium pumilum*, LpPsbP, Saline-Alkali resistance, reactive oxygen species (ROS), jasmonic acid (JA), FoxO

## Abstract

(1) Background: The growth of plants is impacted by salinity and alkali, *Lilium pumilum* (*L. pumilum*) is an ornamental plant with strong resistance to salinity and alkali, while the *LpPsbP* gene is helpful to fully understand the Saline-Alkali tolerance of *L. pumilum*. (2) Methods: Gene cloning, bioinformatics analysis, expression of fusion protein, determination of physiological indices of plant after Saline-Alkali stress, yeast two-hybrid screening, luciferase complementation assay, chromosome walking to obtain the promoter sequence, and then analyzed by PlantCARE. (3) Results: The *LpPsbP* gene was cloned and the fusion protein was purified. The transgenic plants had higher Saline-Alkali resistance than the wild type. A total of eighteen proteins interacting with LpPsbP were screened, and nine sites in the promoter sequence were analyzed. (4) Conclusion: Under Saline-Alkali or oxidative stress, *L. pumilum* will promote the expression of LpPsbP, which will then directly scavenge reactive oxygen species (ROS) in order to protect its photosystem II, reduce its damage, and thus improve the Saline-Alkali resistance of the plant. Moreover, according to some of the literature and the following experiments, two additional speculations are developed on the mechanisms of how two newly found objects, namely jasmonic acid (JA) and FoxO protein, could be involved in ROS scavenging processes were made.

## 1. Introduction 

Saline-Alkali soil not only restricts plant growth, but also leaves potential hidden risks for maintaining ecosystem stability [1]. In China, the proportion of salinized and alkalized soil areas is relatively high, especially in the northeast. For example, a large proportion of the grassland in the Songnen Plain of northeast China has been salinized and alkalized mainly by sodium carbonate (Na_2_CO_3_) and sodium bicarbonate (NaHCO_3_) [2]. Therefore, it is of great importance to conduct studies on Saline-Alkali-tolerant plants.

*Lilium pumilum* (*L. pumilum*) is not only valuable for having the beautiful bright red ornamental flowers, but also for its strong resistance to disease, cold, drought and salinity, which makes it an unparalleled plant material in Saline-Alkali stress research [3,4,5]. *Lilium pumilum* in this study grows naturally in the heavily salinized and alkalized meadow of Daqing suburb of the Songnen Plain with soil pH as high as 9–10. Exploring the molecular interactions of important Saline-Alkali tolerance genes of *Lilium pumilum* can provide more theoretical bases for the Saline-Alkali tolerance traits of the plant.

Plants, algae and cyanobacteria all possess photosystem II (PS II) complexes, which have the unique ability to utilize light energy to oxidize water into molecular oxygen. The PS II complex is composed of more than 20 proteins, including intrinsic and extrinsic membrane subunits. On the thylakoid cavity side of PS II, there are three inorganic ion clusters responsible for catalyzing water oxidation, namely manganese (Mn), calcium (Ca) and chloride (Cl) ion clusters (together called Mn_4_–Ca–Cl_x_ cluster). The Mn_4_–Ca–Cl_x_ cluster in PS II is surrounded by several luminal subunits that stabilize these ionic components. Together with the loop regions of several intrinsic membrane subunits, such as D1, D2, CP43 and CP47, these exogenous membrane subunits support the proper assembly and stabilization of the Mn_4_–Ca–Cl_x_ cluster [6]. This complex is called the oxygen evolving complex (OEC), and the luminal subunits of PS II are called the OEC proteins.

The OEC of higher plants consists of three external proteins with apparent molecular weights of 33, 24 and 17 kDa, which are called PsbO (the PS II subunit O), PsbP (the PS II subunit P) and PsbQ (the PS II subunit Q), respectively [7]. These three extrinsic components interact with intrinsic membrane proteins to form a fully functional oxygen-evolving complex. A group of PsbP family proteins was found in the higher plants. This group includes two PsbP proteins (PsbP1 and PsbP2), two PsbP-like proteins (PPL1 and PPL2) and seven PsbP domain proteins (PPD1–PPD7) [8]. 

The in vivo function of PsbP was first characterized in the green alga *Chlamydomonas reinhardtii*. A very small amount of PsbP may support the substantial accumulation of PS II complexes, whereas higher concentrations of PsbP in the thylakoid lumen are required to maintain the activeness of manganese ion clusters in vivo [9]. In higher plants, the in vivo function of the PsbP protein was first characterized in transgenic tobacco. PsbP–RNAi plants exhibited growth retardation, light green leaf color and low PS II activity. These studies suggest that PsbP is essential for maintaining the activity of PS II in vivo [10]. Over the last 30 years, a significant amount of work has shown that the PsbP protein can increase the rate of oxygen release at physiological calcium and chloride concentrations in green algae and higher plants [11].

In recent years, there have been numerous studies about the relationship between PsbP protein and stresses. The expression level of PsbP was increased under chilling stress and the application of uniconazole, indicating that low-temperature stress promoted the expression of PsbP [12]. A high-temperature environment greatly inhibited the expression of essential genes involved in the photosynthetic pathway, including PsbP [13]. It was also found that the expression of *PsbP* was up-regulated under dehydration stress, suggesting that PsbP may play an important functional role in drought tolerance in chickpeas [14]. However, studies directly about the relationship between PsbP protein and Saline-Alkali resistance are still limited. The arbuscular mycorrhizal fungus (AMF) was inoculated into *Vaccinium corymbosum*, and experiments under alkaline stress were conducted, finding that the increase in *PsbP* expression in the experimental group promoted the ability of AMF-inoculated plants to resist alkali stress [15].

The principal site of reactive oxygen species (ROS) production in chloroplast thylakoids is the reaction center of PS II [16]. The control of production and removal of ROS in chloroplasts is of great importance in promoting the plants’ salinity tolerance. The PsbP protein plays an important role in maintaining the activity of PS II to release oxygen.

*L. pumilum* is a Saline-Alkali-tolerant and adaptable plant. Considering all the reviewed literature, information and real situations above, we made a hypothesis according to which the *LpPsbP* gene is related to the Saline-Alkali resistance and ROS removal of *L. pumilum*, so the LpPsbP protein may be involved in the regulatory pathway of the Saline-Alkali resistance of *L. pumilum* by scavenging ROS. In this study, the *LpPsbP* gene was cloned and its relationship with other species was analyzed using bioinformatics methods. *LpPsbP* was transferred into tobacco to observe the stresses tolerance, LpPsbP interacting proteins were screened by yeast two-hybrid and the promoter of *LpPsbP* was analyzed using PlantCARE. The results show that both the experimental conclusions drawn under Saline-Alkali stress conditions and the properties of the screened interacting proteins were related to ROS. Finally, the hypothesis according to which LpPsbP can confer Saline-Alkali resistance to the plants by scavenging ROS was proved. Moreover, according to some studies and the following experiments, two additional speculations on the mechanism of how two newly found two objects, namely jasmonic acid (JA) and FoxO protein, could be involved in ROS scavenging processes were made. This paper attempts to investigate the relationship between LpPsbP and Saline-Alkali stresses by means of molecular biology, genetics and plant physiology methods. It lays a foundation for revealing the molecular mechanism and functional analysis of the *L. pumilum* gene family in response to Saline-Alkali stress, and has certain scientific significance for making full use of Saline-Alkali soil resources.

## 2. Results

### 2.1. Cloning of Open Reading Frame of LpPsbP Gene

Using *L. pumilum* cDNA as a template and *LpPsbP-F* and *LpPsbP-R* as primers (shown in Table 1), a DNA band with the size of about 800 bp was obtained by PCR, and the result of sequencing from a biotechnology company was 795 bp. 

### 2.2. Genetic Relationship between PsbP of L.pumilum and PsbP of Other Species

**Figure 1:**
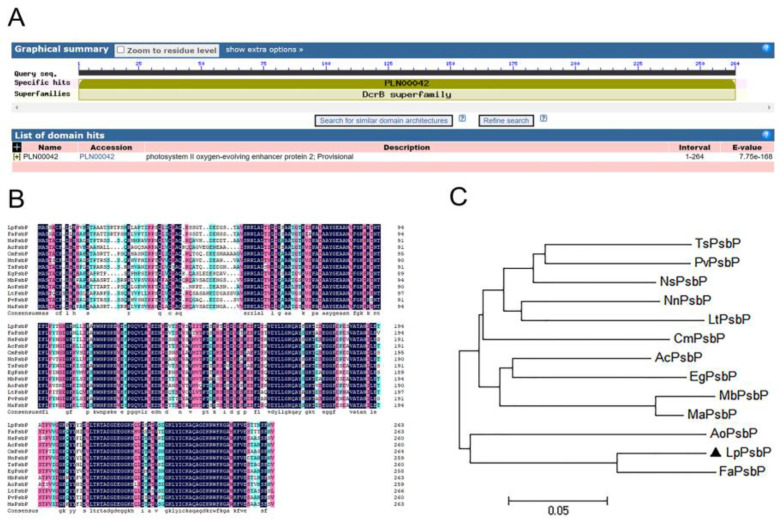
Bioinformatics analysis of LpPsbP. (**A**): Conservative domain analysis of LpPsbP using NCBI. Result shows that there is a highly conserved DcrB domain in LpPsbP. (**B**): Amino acid sequence alignment of LpPsbP with other homologous proteins of different plant species. The amino acid sequence of this transcript was similar to that alignment of the PsbP amino acid sequence from other species: *Fritillaria agrestis*, FaPsbP, O49080.1, 89.77%; *Nyssa sinensis*, NsPsbP, KAA8524123.1, 75.46%; *Ananas comosus*, AcPsbP, XP_020096384.1, 75.09%; *Cinnamomum micranthum F. Kanehirae*, CmPsbP, RWR81876.1, 73.98%; *Nelumbo nucifera*, NnPsbP, XP_010247826.1, 73.64%; *Tetracentron sinense*, TsPsbP, KAF8408713.1, 75.46%; *Elaeis guineensis*, EgPsbP, XP_010906959.1, 75.00%; *Musa balbisiana*, MbPsbP, THU61210.1, 77.53%; *Asparagus officinalis*, AoPsbP, XP_020256197.1, 73.23%; *Ipomoea triloba*, LtPsbP, XP_031126809.1, 75.75%; *Pistacia vera*, PvPsbP, XP_031257256.1, 73.23%; *Musa acuminata*, MaPsbP, XP_009407699.1, 76.78%. (**C**): Phylogenetic tree analysis of LpPsbP and its homologous proteins. The scale bar represents that there is a 5% difference in this given length. The related species are shown in (**B**).

The amino acid sequence of LpPsbP was predicted by conserved domains using NCBI, finding that LpPsbP has a highly conserved DcrB domain at amino acid positions 1-792, which belongs to the DcrB superfamily (Figure 1A).

By BLAST analysis in the NCBI database, the amino acid sequence of PsbP in *L. pumilum* has a high similarity with that of other plants. DNAMAN software was used to compare the amino acid sequence of the PsbP cloned from *L. pumilum* with that of PsbP of other species, finding that the amino acid sequence of the PsbP protein of *L. pumilum* had a high homology with that of other plants (Figure 1B). Therefore, the gene cloned from *L. pumilum* cDNA was considered as PsbP and named as LpPsbP.

Based on the comparison of amino acid sequences and phylogenetic relationships, the phylogenetic tree was constructed using the MEGA_64 program, which shows that the PsbP protein is closely related to the PsbP of *A. officinalis* and *F. agrestis* (Figure 1C).

### 2.3. Expression Characteristics of LpPsbP Gene in the Leaves of L. pumilum after Stress Treatment

**Figure 2:**
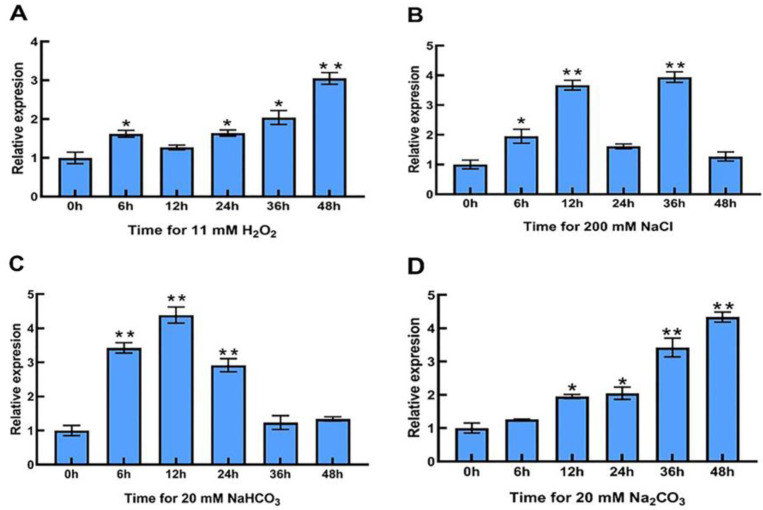
Expression characteristics of *LpPsbP* gene in the leaves of *L. pumilum* after stress treatment. The RNA in the leaves was extracted and then reverse-transcribed into cDNA and the levels of relative expression are gained through qRT-PCR. “*” indicates significant difference, Student’s *t*-test (P<0.05, ±SEM). “**” indicates extremely significant difference. (**A**): Expression of *LpPsbP* gene at 11 mM H_2_O_2_ treatment at different times. (**B**): Expression levels of *LpPsbP* gene at 200 mM NaCl treatment at different times. (**C**): Expression levels of *LpPsbP* gene at 20 mM NaHCO_3_ treatment at different times. (**D**): Expression levels of *LpPsbP* gene at 20 mM Na_2_CO_3_ treatment at different times.

After different times of H_2_O_2_ stress, the expression of *LpPsbP* in the leaves of *L. pumilum* was up-regulated about 1.5 times at 6 h, decreased slightly at 12 h and then continued to rise, until the expression reached the peak value of 3.05 times at 48 h (Figure 2A).

After NaCl stress for different times, the expression of *LpPsbP* in the leaves of *L. pumilum* increased continuously at first, then decreased dramatically and then surged to the maximum before dropping to the lowest, but the expression was still slightly higher than that in the control group. The expression of the *LpPsbP* gene peaked at 3.93 times after 36 h of NaCl treatment (Figure 2B).

After different times of NaHCO_3_ stress, the expression of *LpPsbP* in the leaves of *L. pumilum* climbed first and then decreased with the increase in treatment time. The *LpPsbP* gene expression peaked at 4.38 times after 12 h of NaHCO_3_ stress (Figure 2C).

At different times of Na_2_CO_3_ stress, the expression of *LpPsbP* in the leaves of *L. pumilum* raised continuously with the increase in treatment time. The expression of *LpPsbP* reached a peak value of 4.33 times at 48 h after Na_2_CO₃ treatment (Figure 2D).

### 2.4. Optimization Conditions for Induction and Purification of Recombinant pGEX-LpPsbP

**Figure 3:**
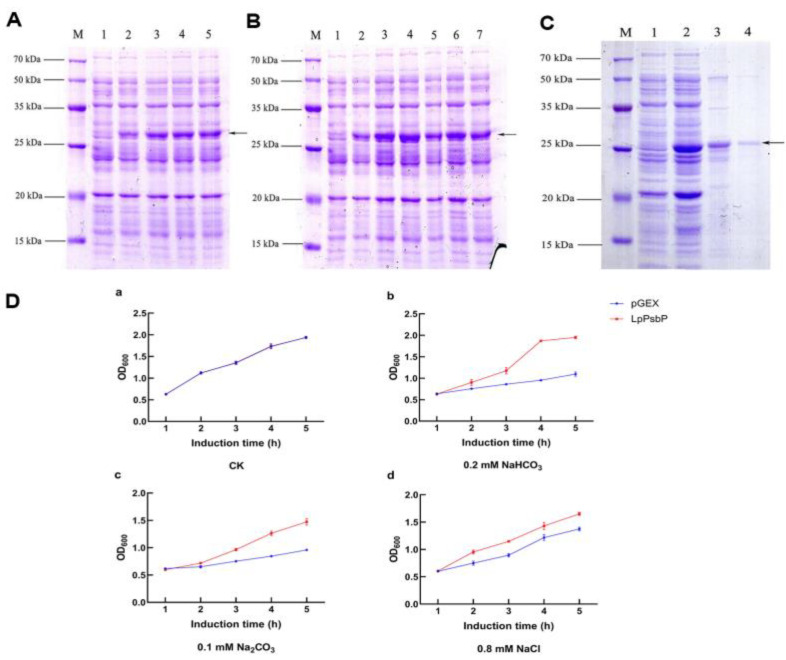
Protein induction experiment. (**A**): pGEX-LpPsbP induction at different IPTG concentrations (M: protein marker; 1, 2, 3, 4, 5: the expression of LpPsbP protein was induced by 0, 0.5, 1, 1.5, 2 mM IPTG); (**B**): pGEX-LpPsbP induction at different times (M: protein marker; 1, 2, 3, 4, 5, 6, 7: the expression of LpPsbP protein was induced for 0, 0.5, 1, 2, 3, 4, 5 h, respectively). (**C**): pGEX-LpPsbP protein purification using purification column with GST purification resin. M: protein marker; 1: induced without IPTG; 2: induced with 1 mM IPTG for 5 h; 3: pGEX-LpPsbP renatured protein; 4: pGEX-LpPsbP purified protein. (**D**): Growth curves of *Escherichia coli* under different Saline-Alkali stresses. a: The OD_600_ values of pGEX and LpPsbP induced for 5 h under untreated conditions (CK). b: The OD_600_ value of bacterial solution after 5 h induction by pGEX and LpPsbP under the treatment of 0.2 mM NaHCO_3_. c: The OD_600_ value of bacterial liquid after 5 h induction by pGEX and LpPsbP under the treatment of 0.1 mM Na_2_CO_3_. d: The OD_600_ value of bacterial solution after 5 h induction by pGEX and LpPsbP under the treatment of 0.8 mM NaCl.

After pGEX-LpPsbP was transferred into BL21-competent cells, the activated bacterial solution was added with 0 mM, 0.5 mM, 1 mM, 1.5 mM and 2 mM of isopropyl-beta-D-thiogalactopyranoside (IPTG), and induced at 37℃ for 5 h. The LpPsbP protein first increased at 0.5 mM IPTG. After 1 mM IPTG, the expression of LpPsbP tended to be stable and did not increase (Figure 3A).

Another seven activated bacterial solutions were added with 1 mM IPTG and induced at 37℃ for 0 h, 0.5 h, 1 h, 2 h, 3 h, 4 h and 5 h. The LpPsbP began to express after 0.5 h of induction and the expression level reached the maximum value 1 h after induction (Figure 3B).

In order to obtain the high purity protein of pGEX-LpPsbP, the lysed protein was purified in a purification column with glutathione S-transferase (GST) purification resin. After 1 h of gentle shaking, the filtrate “pGEX-LpPsbP refolding protein” was obtained and eluted twice with protein cleaning buffer. Then, “pGEX-LpPsbP purified protein” was obtained by eluting the protein eluent with buffering solution twice. Finally, the result of SDS-PAGE showed that the target band was successfully purified (Figure 3C).

### 2.5. Resistance Analysis of Bacterial Solution Expressing LpPsbP Protein under Different Saline-Alkali Stresses

The overexpression of the LpPsbP protein promoted bacterial growth in the solution under different Saline-Alkali stresses. Under normal culture conditions, the optical density in 600 nm (OD_600_) values of bacterial solution after 5 h of induction using pGEX and LpPsbP were both 1.94 (Figure 3D(a)). In the presence of 0.2 mM NaHCO_3_, the OD_600_ values of bacterial solution after 5 h of induction using pGEX and LpPsbP were 1.10 and 1.95, respectively (Figure 3D(b)). Under the treatment of 0.1 mM Na_2_CO_3_, the OD_600_ values of bacterial solution after 5 h of induction using pGEX and LpPsbP were 0.96 and 1.47, respectively (Figure 3D(c)). In the presence of 0.8 mM NaCl, the OD_600_ values of bacterial solution after 5 h of induction with pGEX and LpPsbP were 1.03 and 1.65, respectively (Figure 3D(d)). 

### 2.6. Gene Expression Analysis of pBI121-LpPsbP Overexpressing Tobaccos

**Figure 4:**
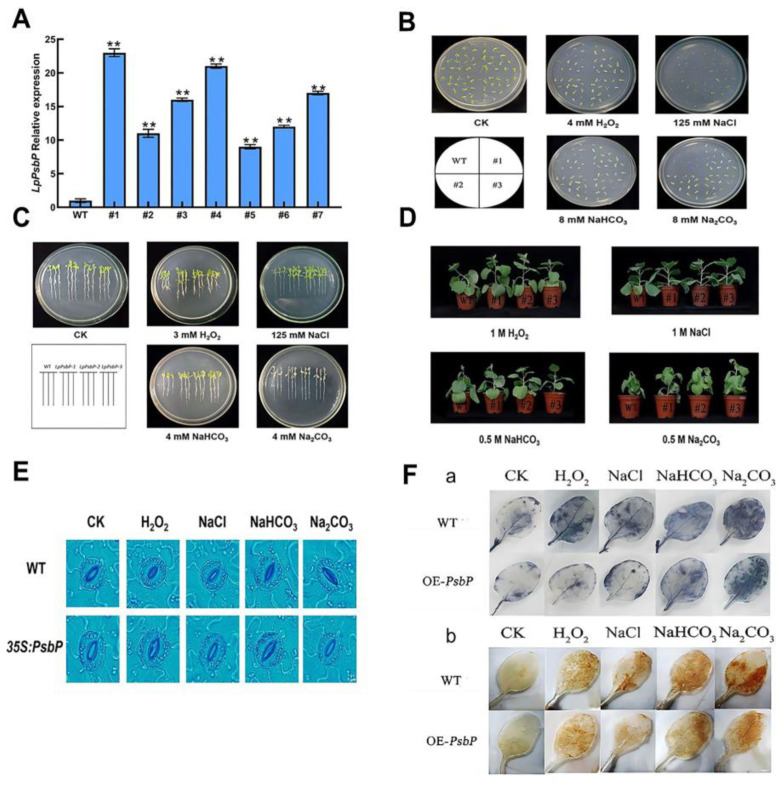
Phenotypic analysis of *LpPsbP* overexpressed tobaccos. (**A**): qPCR identification of transgenic tobacco overexpressing *LpPsbP* gene. cDNA was obtained from the leaves of wild-type and transgenic tobacco plants by reverse-transcribing from RNA. The expression levels were detected by qPCR. WT: wild line; #1-#7: *LpPsbP* transgenic line; “**” indicates extremely significant difference. (**B**): Germination phenotypic analysis of wild-type tobaccos and *LpPsbP* transgenic tobaccos under salt stress. Tobacco seeds from the T3 generation, both wild-type and *LpPsbP* transgenic, were taken and cultured for 7 days in 1/2 MS medium and 1/2 MS media supplemented with 4 mM H_2_O_2_, 125 mM NaCl, 8 mM NaHCO_3_ and 8 mM Na_2_CO_3_, respectively. (**C**): Seeding growth of *LpPsbP* transgenic plants under salt stress. Wild-type tobacco seeds and T3 generation *LpPsbP* transgenic plants were sown separately in control group and groups in 1/2 MS medium supplemented with 3 mM H_2_O_2_, 125 mM NaCl, 4 mM NaHCO_3_ and 4 mM Na_2_CO_3_. The results were recorded after 14 days. (**D**): Phenotypic analysis of *LpPsbP* transgenic plants under salt stress. Plants were irrigated with 20 mL of 1 M H_2_O_2_, 1 M NaCl, 0.5 M NaHCO_3_ and 0.5 M Na_2_CO_3_ solutions for 7 days. (**E**): Stomatal size of wildtype and *LpPsbP* transgenic tobacco under different salt stresses. It can be seen from this figure that after treatment with 4 mM H_2_O_2_, 125 mM NaCl, 8 mM NaHCO_3_ and 8 mM Na_2_CO_3_, stomatas in WT plants obviously closed, while *LpPsbP* transgenic plants only displayed slight stomata closure. (**F**): NBT and DAB staining of various *LpPsbP*-related genetic materials under salt stress. In situ NBT and DAB staining methods were used to detect the accumulation of O^2−^ and H_2_O_2_ in seedling leaves, which reflected the ability of LpPsbP to reduce ROS content in plants. The deeper the blue color is, the more O^2−^ accumulated. The darker the brown color is, the more H_2_O_2_ is accumulated.

The successfully constructed pBI121-LpPsbP plant expression vector was transformed into tobacco plants. The gene expression of *LpPsbP* transgenic tobacco was detected by qPCR. As shown in Figure 4A, the expression of *LpPsbP* in #1-#7 was higher than that in the wild type (WT), among which #1, #4 and #7 had higher expression levels, which were 23.21, 20.20 and 17.7 times those of the WT, respectively. Therefore, the #1, #4 and #7 plants were selected for subsequent experiments.

### 2.7. Phenotypic Analysis of Tobacco with pBI121-LpPsbP Overexpression under Saline-Alkali Stress

#### 2.7.1. Effect of Salt Stress on Seed Germination

In order to investigate the effects of salt stress on the germination of WT seeds and *LpPsbP* transgenic seeds, wild-type and *LpPsbP* transgenic T3 generation tobacco seeds were taken and cultured in 1/2 MS medium and 1/2 MS medium supplemented with 4 mM H_2_O_2_, 125 mM NaCl, 8 mM NaHCO_3_ and 8 mM Na_2_CO_3_, respectively, for 7 days. The observation results are shown in Figure 4B. All seeds germinated in 1/2 MS medium, and the seedlings were green. There was no significant difference between wild-type and *LpPsbP* transgenes visually. Under salt stress of 4 mM H_2_O_2_, 125 mM NaCl, 8 mM NaHCO_3_ or 8 mM Na_2_CO_3_, the germination of wild-type seeds was significantly inhibited and most seeds did not germinate, while the young seedlings of germinated seeds were small and yellow in color. All the transgenic seeds germinated under salt stress, and the seedlings were green and grew larger, which was better than the wild type. These results indicated that *LpPsbP* transgenic seeds had greater tolerance to salt stress during germination.

#### 2.7.2. Analysis of Seedling Resistance of Transgenic Plants under Salt Stress

In order to investigate the resistance analysis of transgenic plants under salt stress, wild-type tobacco seeds and T3 generation *LpPsbP* transgenic plants were sown separately in the control group, groups in 1/2 MS medium supplemented with 3 mM H_2_O_2_, 125 mM NaCl, 4 mM NaHCO_3_ and 4 mM Na_2_CO_3_. The results obtained after 14 days are shown in Figure 4C. In the control medium, wild-type and transgenic seedlings presented green leaves, with long roots and uniform leaves, and there was no significant difference in growth potential between wild-type and transgenic seedlings. In 3 mM H_2_O_2_ medium, wild-type seedlings grew less than transgenic seedlings, and all seedlings had shorter roots. In 125 mM NaCl medium, wild-type and transgenic seedlings had the same growth condition of leaves, and the root length was smaller than that of transgenic seedlings. In 4 mM NaHCO_3_ medium, both wild-type seedlings and transgenic seedlings were yellow, and the root length of the wild-type seedlings was still smaller than that of the wild-type seedlings. In 4 mM Na_2_CO_3_ medium, the leaves of the wild-type seedlings and transgenic seedlings developed less, and the leaves of wild-type leaves were completely yellow and transparent, while some parts of the *LpPsbP* transgenic leaves remained green. The results show that the *LpPsbP* gene affected seedlings under salt stress and enhanced their resistance to salt stress.

#### 2.7.3. Resistance Analysis of Transgenic Plants under Salt Stress

In order to study the resistance analysis of transgenic plants under salt stress, WT and transgenic tobacco plants with the same growth size in the pot were selected and irrigated with 20 mL of 1 M H_2_O_2_, 1 M NaCl, 0.5 M NaHCO_3_ and 0.5 M Na_2_CO_3_ solutions for 7 days. The growth phenotype (Figure 4D) was observed. Under 1 M H_2_O_2_ treatment, wild-type plants appeared to bend, while transgenic plants remained upright. Under the treatment of 1 M NaCl and 0.5 M NaHCO_3_, the plants had no tendency to bend but compared with the transgenic plants, the first to the fourth leaves of WT plants were drooping. Under 0.5 M Na_2_CO_3_ treatment, the leaves of wild-type and transgenic plants were drooping from bottom to top, compared with that of wild transgenic plants, which also demonstrated stem bending. The results show that *LpPsbP* transgenic plants were more tolerant to salt stress than WT plants.

#### 2.7.4. Stomatal Opening in WT and *LpPsbP* Transgenic Plants after Salt Stress

The stomatal opening and closing of WT and *LpPsbP* transgenic plants after salt stresses were observed under a microscope. There is no significant difference in stomatal size between WT plants and transgenic plants without salt stress treatment (Figure 4E). After treatment with 4 mM H_2_O_2_, 125 mM NaCl, 8 mM NaHCO_3_ and 8 mM Na_2_CO_3_, the WT plants exhibited visible stomatal closure, while the *LpPsbP* transgenic plants only exhibited slight stomata closure. These results indicate that the WT plants were more sensitive to stomatal switching after salt stress than transgenic plants.

#### 2.7.5. Assessment of O^2−^ and H_2_O_2_ Accumulation in Tobacco by NBT and DAB Staining

In order to further verify the content of O^2−^ and H_2_O_2_ in tobacco plants, nitro-blue tetrazolium (NBT) and diaminobenzidine (DAB) staining methods were utilized to detect O^2−^ and H_2_O_2_ in seedling leaves *in situ*, so as to understand the ability of LpPsbP to reduce ROS content in plants. The deeper the blue color is, the more O^2−^ is accumulated. The darker the brown color is, the more H_2_O_2_ is accumulated (Figure 4F). The results illustrate that the staining results of NBT and DAB show no significant difference between wild-type and transgenic plants under the control condition. The staining deepened after salt stress treatment, and the staining of wild-type leaves was deeper than that of transgenic leaves, indicating that the overexpression of the *LpPsbP* gene can reduce the accumulation of ROS in plants under salt stress. The *LpPsbP* gene can enhance plant resistance under salt stress.

### 2.8. Analysis of Physiological Indices of Tobacco under Saline-Alkali Stress

#### 2.8.1. Relationship between *LpPsbP* Gene and Gas Exchange Parameters in Tobacco Leaves

**Figure 5:**
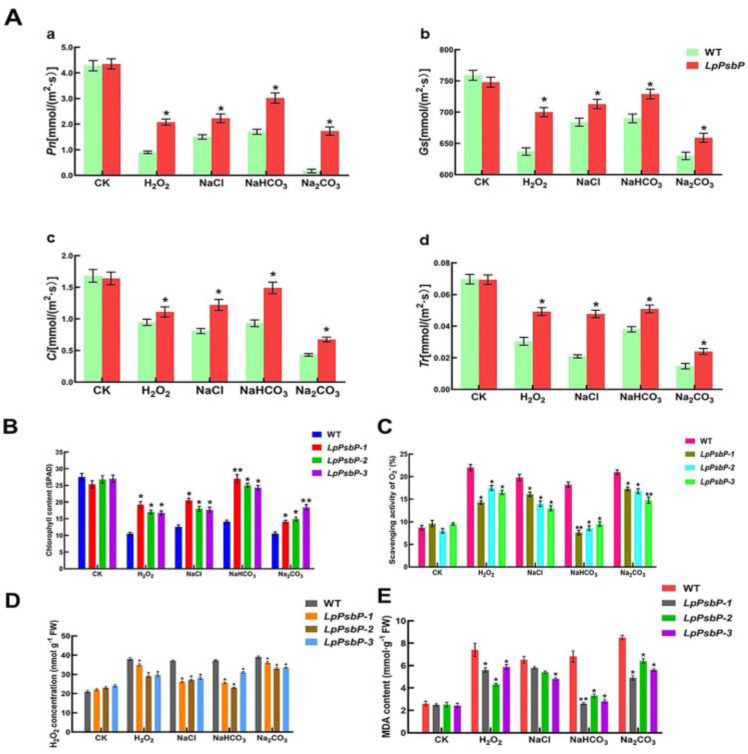
Analysis of physiological indices of tobacco under Saline-Alkali stress. “*” indicates significant difference, Student’s *t*-test (P<0.05, ±SEM). “**” indicates extremely significant difference. (**A**): Gas exchange parameters of *LpPsbP* tobacco under salt stress. LI-6400 photosynthometer was used to measure the following indices. (**a**): Net photosynthetic rate (*P*n), (**b**): Stomatal conductance (*G*s), (**c**): Intercellular CO_2_ (*C*i), (**d**): Transpiration rate (*T*r) of wild-type and *LpPsbP* transgenic tobacco under different salt treatments (1 M H_2_O_2_, 1 M NaCl, 0.5 M NaHCO_3_ and 0.5 M Na_2_CO_3_). (**B**): Determination of chlorophyll content in *LpPsbP* tobacco under salt stress. SPAD chlorophyll meter was used to measure the chlorophyll contents of tobacco under different salt stresses (125 mM NaCl, 8 mM Na_2_CO_3_, 8 mM NaHCO_3_ and 4 mM H_2_O_2_). (**C**): Determination of O^2−^ content in *LpPsbP* tobacco under salt stress (125 mM NaCl, 8 mM Na_2_CO_3_, 8 mM NaHCO_3_ and 4 mM H_2_O_2_). Oxidative damage and O^2−^ accumulation in plants are both consequences of salt stress. By evaluating the O^2−^ content in transgenic plants, it was possible to evaluate the capacity of LpPsbP to reduce ROS content in tobacco. (**D**): Determination of H_2_O_2_ content in *LpPsbP* tobacco under salt stress. An H_2_O_2_ kit (Grace, Suzhou, China) was used to measure the H_2_O_2_ content of wild-type and transgene plants under controlled and salt stress (125 mM NaCl, 8 mM Na_2_CO_3_, 8 mM NaHCO_3_ and 4 mM H_2_O_2_). (**E**): Determination of MDA content in *LpPsbP* tobacco under salt stress. The levels of malondialdehyde (MDA) produced by several tobacco lines when exposed to salt stress (125 mM NaCl, 8 mM Na_2_CO_3_, 8 mM NaHCO_3_ and 4 mM H_2_O_2_) were examined to evaluate the relationship between excessive ROS accumulation and cell damage.

In order to explore an association between the *LpPsbP* gene and leaf gas exchange parameters in tobacco, a LI-6400 photosynthometer was used to measure the net photosynthetic rate (*P*n), stomatal conductance (*G*s), intercellular CO_2_ (*C*i) and transpiration rate (*T*r) of wild-type and *LpPsbP* transgenic tobacco under different salt treatments (1 M H_2_O_2_, 1 M NaCl, 0.5 M NaHCO_3_ and 0.5 M Na_2_CO_3_). There was no significant difference in gas exchange parameters between wild-type and transgenic lines under normal conditions. Under salt stress processing, wild-type lines and transgenic lines in tobacco leaf net photosynthetic rate, transpiration rate and stomatal conductance, as well as intercellular CO_2_, decreased, but these various parameters of transgenic lines are higher than those of the wild type (Figure 5A). Thus, the overexpression of the *LpPsbP* gene can effectively alleviate the reduction in gas exchange function in leaves induced by salt stress.

#### 2.8.2. Measurement of Chlorophyll Content of Tobacco under Different Salt Stresses

The chlorophyll contents of tobacco under different salt stresses were measured by a SPAD chlorophyll meter (Figure 5B). The chlorophyll content of wild-type and transgenic tobacco plants in the blank control group was similar when the leaves of the same part were taken from top to bottom. The chlorophyll content of plants was significantly decreased after salt treatment, but the chlorophyll content of transgenic plants was higher than that of the wild-type plants under different stresses. *LpPsbP*-overexpressing plants had higher salinity tolerance than wild-type plants.

#### 2.8.3. Measurement of O^2−^ Content in Transgenic Plants

Salt stress can lead to the accumulation of O^2−^ in plants and oxidative damage. The ability of LpPsbP to reduce ROS content in tobacco was estimated by measuring O^2−^ content in transgenic plants. Under normal conditions, O^2−^ content in normal tobacco and transgenic tobacco did not change significantly. After salt stress, O^2−^ content in tobacco leaves began to differ. After salt stress, the O^2−^ content in leaves increased significantly, while the O^2−^ content in transgenic lines was lower than that of wild-type lines (Figure 5C), which was consistent with the results of NBT staining for superoxide anion (Figure 4F). These results indicate that the presence of the *LpPsbP* gene inhibit the accumulation of superoxide anion in tobacco leaves.

#### 2.8.4. Measurement of H_2_O_2_ Content in Transgenic Plants

In order to determine the effect of the *LpPsbP* gene on the accumulation of reactive oxygen species under salt stress, the H_2_O_2_ content of wild-type and transgene plants under controlled and salt stress was measured using an H_2_O_2_ kit (Grace, Suzhou, China) (Figure 5D). The content of H_2_O_2_ in wild-type and transgenic lines is similar without salt stress, and they are both low. The content of H_2_O_2_ in the plants under salt stresses, and the content of H_2_O_2_ in the wild-type lines was higher than that in the transgenic lines, which was consistent with the result of DAB staining. These results indicate that the wild-type lines accumulated more H_2_O_2_ under salt stress and were more sensitive to salt stress. The transgenic plants accumulated less H_2_O_2_ and were resistant to salt stress.

#### 2.8.5. Measurement of Malondialdehyde Content Produced by Different Tobacco Lines under Salt Stress

In order to determine the association between high ROS accumulation and cell damage, we examined the malondialdehyde (MDA) content produced by different tobacco lines under salt stress (Figure 5E). The wild-type lines with higher ROS levels accumulate more MDA, while transgenic trains with lower ROS content also had lower MDA expressions. The salt stresses can cause a large accumulation of reactive oxygen species in plants, and the presence of the *LpPsbP* gene can reduce the accumulation of reactive oxygen species and reduce plant damage.

### 2.9. Screening and Cotransformation Validation of cDNA Libraries

pGBKT7-LpPsbP was successfully constructed by using two restriction sites of BamH1 and Sal1. The transformed pGBKT7-LpPsbP was fused with the cDNA library of *L. pumilum*, and the DNA of 19 blue strains was extracted for PCR detection. The results show that only No. 5 had no evident band, and the remaining 18 samples were all given a single band, which was sequenced. The homology of these sequences was outputted in NCBI. The repeated sequences were removed by comparison, and five sequences related to Saline-Alkali resistance were obtained. The protein codes are listed in Table 2.

**Figure 6:**
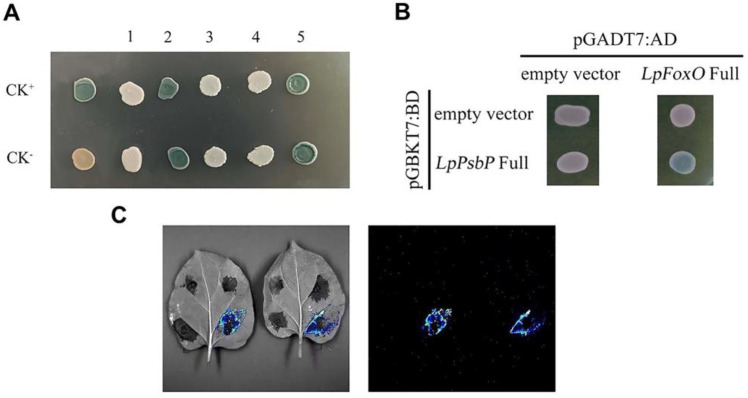
Validation of LpPsbP interacting proteins. (**A**): cDNA library screening of pGBKT7-LpPsbP. Y2H was cotransformed successfully with 5 plasmids related to salt stress and pGBKT7-LpPsbP. When the yeast was grown on SD-Trp-Leu-His plus X-gal media at 30 °C, the transformed yeasts with plasmids 2 and 5 grew as colonies and became blue, while the yeasts with plasmids 1, 3 and 4 did not. (**B**): pGBKT7-LpPsbP and pGADT7-LpFoxO co-conversion validation. On SD-Trp-Leu-His plus X-gal media, the strains cotransformed with pGBKT7-LpPsbP and pGADT7-LpFoxO were able to establish blue colonies, whereas the control group was unable to do that. (**C**): Luciferase complementation assay. The first quadrant: both NLuc and CLuc were unloaded, the second quadrant: NLuc was unloaded, the third quadrant: CLuc was unloaded and the fourth quadrant: pCAMBIA1300-NLuc-PsbP and pCAMBIA1300-CLuc-FoxO.

A total of five successfully compared plasmids related to salinity stress and pGBKT7-LpPsbP plasmid were cotransformed into Y2H and cultured on SD-Trp-Leu-His plus X-α-gal medium at 30℃. The plasmid 2 and plasmid 5-transformed yeasts grew as colonies on the medium and turned blue, while plasmids 1, 3 and 4 did not turn blue. It can be preliminarily concluded that No. 2 and No. 5 interact with pGBKT7-LpPsbP (Figure 6A). In this experiment, the No. 2 *LpFoxO* gene was selected for subsequent cloning and verification.

For further verification, pGADT7 and *LpFoxO* plasmids with added restriction site were digested with BamH1 and Xho1, and pGADT7-LpFoxO was successfully constructed. The strains cotransformed with pGBKT7-LpPsbP and pGADT7-LpFoxO could grow blue colonies on SD-Trp-Leu-His plus X-α-gal medium, while there were no blue colonies growth in the control group (Figure 6B). These results indicate that LpPsbP and LpFoxO proteins can interact with each other.

### 2.10. Luciferase Complementation Assay

After the control groups (functional fragment of N-terminus luciferase, NLuc and C-terminus luciferase, CLuc empty load, NLuc empty load and CLuc empty load) and the experimental group (pCAMBIA1300-NLuc-PsbP and pCAMBIA1300-CLuc-FoxO) were transferred into *Agrobacterium tumefaciens* GV1301, they were then successively transferred into four quadrants of the same leaf (I, II, III, IV), in which the experimental group was located in the fourth quadrant. As shown in Figure 6C, the experimental group emits visible fluorescence, indicating that PsbP interacts with FoxO.

### 2.11. Analysis of LpPsbP Promoter

**Figure 7:**
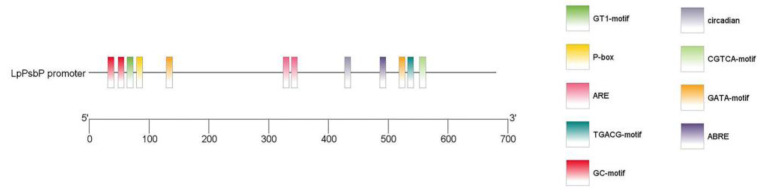
Visual analysis of the *LpPsbP* promoter. The acquired promoter sequences underwent visual analysis using the TBtools software to present some cis-acting elements such as GT1-motif and P-box. Sequence analysis revealed that this sequence comprised numerous responsive and regulatory regions.

Through three rounds of PCR amplification by the chromosome walking method, a 680 bp fragment was cloned. The cis-acting element of the *LpPsbP* promoter sequence was analyzed using PlantCARE (shown in Table 3). Then, the TBtools software (Guangzhou, China) was used to carry out a visual analysis of the obtained promoter sequences (Figure 7). The analysis of sequence indicated that this sequence contained multiple regulatory and responsive sites.

## 3. Discussion

There are many stress factors affecting plant growth and survival, among which Saline-Alkali stress is one of the main abiotic stresses. Saline-Alkali stress can lead to the accumulation of ROS, destroy enzymes in cells and cause oxidative damage to membranes [22]. The PS II subunit P (PsbP) protein is a major component of the oxygen evolution complex (OEC) of chloroplast PS II. The function of the PsbP protein was first proved in transgenic tobacco. RNAi-inhibited PsbP mutants exhibited a severe growth inhibitory phenotype in tobacco, suggesting that PsbP is essential for maintaining PS II activity in vivo [10]. At present, there are many studies on the structural and functional characteristics of plant PsbP, but there are a few studies on the molecular response of PsbP involved in plant stress. The *LpPsbP* gene was cloned from *L. pumilum*. The amino acid sequence alignment between *L. pumilum* and other species showed that it conserved between *L. pumilum* and other species up to 80%. The PsbP protein was closely related to plants such as *F. agrestis* by constructing an evolutionary tree.

There were many studies indicating that Saline-Alkali stress can increase the expression of PsbP. For instance, there was the accumulation in PsbP protein and stronger secondary metabolites biosynthesis ability in plants under high-pH stress [15]. IbPsbP was up-regulated 2.6- and 6-fold by 24 h NaCl treatment and H_2_O_2_ treatment, respectively [23].

The expression of the *LpPsbP* gene was significantly up-regulated in the transcriptome of leaves in *L. pumilum* treated with Saline-Alkali stress. In order to observe whether *LpPsbP* expression was changed with Saline-Alkali stress or not, *L. pumilum* was treated with different stresses (H_2_O_2_, NaCl, NaHCO_3_ and Na_2_CO_3_). The results show that the expression of the *LpPsbP* gene changed as treatments started. After being given 48 h of H_2_O_2_, 36 h of NaCl, 12 h of NaHCO_3_ and 48 h of Na_2_CO_3_ treatment, the expression levels of the *LpPsbP* gene reached the maximum. This showed that LpPsbP may play an important role in regulating the response to abiotic stress in *L. pumilum*.

The LpPsbP–GST fusion protein displayed higher tolerance than the GST protein in *E. coli*. The germination rate of the transgenic plant was significantly higher than that of the wild-type under Saline-Alkali stress, which indicates that the presence of LpPsbP alleviated the damage of stress. Under Saline-Alkali stress, the growth and seedling formation of transgenic seedlings are relatively less inhibited. The physiological parameters, such as net photosynthetic rate (*P*n), stomatal conductance (*G*s), intercellular CO_2_ (*C*i), transpiration rate (*T*r) and chlorophyll content were measured, finding that the tolerances of transgenic lines were higher than those of wild types.

The PsbP was found to be one of the proteins involved in the scavenging mechanisms of reactive oxygen species (ROS) in plants [24]. RXLR31154 was reported to reduce the accumulation of H_2_O_2_ by stabilizing PsbP [25]. PsbP Domain Protein 5 (PPD5) can regulate drought resistance by modulating the accumulation of H_2_O_2_ in guard cells [26]. In order to know whether the higher tolerance of the transgenic lines than that of the wild type was caused by the clearance of ROS, the contents of O^2−^, H_2_O_2_ and malondialdehyde (MDA) of wild-type and transgenic lines under salt stress were analyzed. The analysis of the results (NBT and DAB staining, O^2−^, H_2_O_2_ and MDA content in the leaves of *LpPsbP*-overexpressing tobaccos) showed that the accumulation of O^2−^ and H_2_O_2_ in the leaves of *LpPsbP*-overexpressing plants was lesser than that of wild-type plants. Therefore, it is speculated that the overexpression of the *LpPsbP* gene would enhance the scavenging of reactive oxygen species in plants.

A total of 18 proteins interacting with LpPsbP were screened by the method of yeast two-hybrid. FoxO belongs to forkheadbox (Fox) protein family, a class of transcription factors with wing-like helix structure in the DNA binding region [24]. FoxO is an important regulator of various intracellular processes. Studies have shown that ROS can regulate the activation and gene expression of the O subgroup of the forkhead box transcription factor family at multiple levels [27]. In some cell types (for example, hematopoietic stem cells), ROS was cleared by upregulating the expression of antioxidant enzymes by the forkhead box O (FoxO) family of transcription factors [28]. Moreover, through the transcriptional activation of genes producing ROS-scavenging proteins such as MnSOD and catalase, FoxO proteins also control aging and cellular senescence [29]. In another study, it is found that KDM5 modulates ROS through the interaction with FoxO [30]. Based on this, we speculate that LpPsbP can interact with FoxO to regulate ROS content. 

Additionally, the chromosomal walking method was used to clone the *LpPsbP* promoter gene. Following analysis, we discovered that the promoter sequence had cis-acting elements in response to abiotic stresses (such as anaerobic condition), light, hormones (such as abscisic acid), etc. Additionally, there were two sites involved in the response of methyl jasmonate (MeJA). Signaling pathways of jasmonic acid are related to ROS wave, and specifically, the role of JA mainly involved in suppressing the ROS wave during responses to high light stress or wounding [31]. Therefore, the promoter of the *LpPsbP* can be involved in a variety of biological metabolic pathways.

## 4. Materials and Methods

### 4.1. Cloning of LpPsbP Gene

Trizol method was used to extract total RNA from *L. pumilum*, and the full-length cDNA was obtained by Takara reverse transcription kit (Tokyo, Japan). According to the transcriptome sequencing results of *L. pumilum* bulbs, the open reading frame (ORF) of *LpPsbP* gene sequence was found and SnapGene software (Boston, MA, USA) was used to design the specific primers. All sequences of the primers used in this paper are shown in Table 1. The target gene was amplified by PCR using gene-specific primers (*LpPsbP-F* and *LpPsbP-R*, shown in Table 1) using cDNA as a template. PCR products were sent to Kumei Biotechnology Co., LTD (Changchun, China) for sequencing.

### 4.2. Bioinformatics Analysis

BLAST analysis of LpPsbP was performed on the NCBI website to find other PsbPs with high homology to LpPsbP in other species. The homologous amino acid sequences of proteins were aligned using DNAMAN software (San Ramon, CA, USA), and the phylogenetic tree was constructed using MEGA_64 software to observe the relationship between PsbP of *L. pumilum* and PsbPs of other species. NCBI was used to analyze the conserved domain of PsbP.

### 4.3. Investigating the Expression Characteristics of LpPsbP Gene in the Leaves of L. pumilum after Stress Treatment

The tissue-cultured *L. pumilum* was transferred to MS medium (pH 5.8) without any stress treatment (CK) and MS medium containing 200 mM NaCl, 20 mM NaHCO_3_, 11 mM H_2_O_2_ and 20 mM Na_2_CO_3_. RNA was extracted from leaves after 0 h, 6 h, 12 h, 24 h, 36 h and 48 h of different stresses; then, they were reversely transcribed into cDNA, and the expression of *LpPsbP* gene in *L. pumilum* leaves after stress treatment was analyzed by qRT-PCR using the designed primers (*LpPsbP qPCR-F* and *LpPsbP qPCR-R*, shown in Table 1).

### 4.4. Construction of LpPsbP Protein Fusion Expression Vector and Optimization of Induced Expression Conditions

Primers with BamH1 and Xho1 double restriction sites (*LpPsbP-BamH1-F* and *LpPsbP-Xho1-R*, shown in Table 1) were designed to construct pGEX-LpPsbP prokaryotic expression vector. The successfully constructed plasmid was transferred into protein expressing strain BL21. pGEX-LpPsbP fusion protein was induced by IPTG and the expression conditions were optimized; then, the fusion protein of pGEX-LpPsbP was purified.

*E. coli* strain expressing empty vector pGEX was used as a control to compare the growth trend of strains expressing pGEX-LpPsbP protein under different stress conditions. When the OD_600_ values of the bacterial solution reached about 0.5, different stresses (0.2 mM NaHCO_3_, 0.1 mM Na_2_CO_3_ and 0.8 mM NaCl) were added for different times, and the new OD_600_ values of the bacterial solution expressing pGEX and pGEX-LpPsbP were measured.

### 4.5. Resistance Analysis of LpPsbP Transgenic Tobacco under Saline Stress

The plant expression vector pBI121-LpPsbP was constructed using BamH1 and Xho1. The successfully constructed plasmid was transformed into *A. tumefaciens*-competent cells EH105 by electroshock method, and then pBI121-LpPsbP transgenic tobacco was transformed. The co-cultured leaves were transferred to the screening medium, and seedlings were generated. Finally, DNA of transgenic tobacco seedlings was extracted for PCR identification. After identification, RNA in the leaves of wild-type and transgenic tobacco plants were extracted and reverse-transcribed into cDNA. Finally, qPCR primers were used to detect the expression of transgenic plants.

The seeds of wild-type and T3 *LpPsbP* overexpressed tobacco were sown in the control medium supplemented with 125 mM NaCl, 8 mM Na_2_CO_3_, 8 mM NaHCO_3_ and 4 mM H_2_O_2_ at the same time and cultured for 7 days to observe the germination of seeds. Three groups of repeated biological experiments were performed. 

The T3 seeds of wild-type and *LpPsbP* transgenic lines were simultaneously seeded in the control medium supplemented with 125 mM NaCl, 4 mM Na_2_CO_3_, 4 mM NaHCO_3_ and 3 mM H_2_O_2_, and the plates were placed vertically for the growth of the roots. Thus, the roots were allowed to grow vertically for 14 days to observe the growth phenotype of seedlings at this time. Three groups of repeated biological experiments were performed.

Wild-type and *LpPsbP* transgenic tobacco cultivated in soil with similar size and same growth conditions were selected, respectively. The growth phenotype was observed by watering with 20 mL of 1 M NaCl, 0.5 M Na_2_CO_3_, 0.5 M NaHCO_3_ and 1 M H_2_O_2_ solutions for 7 days. Three groups of repeated biological experiments were performed.

### 4.6. Different Indices of Tobacco with pBI121-LpPsbP Overexpression under Saline-Alkali Stress

The stomata of the same part of the tobacco leaves (WT and *LpPsbP*#1) under 125 mM NaCl, 8 mM Na_2_CO_3_, 8 mM NaHCO_3_ and 4 mM H_2_O_2_ were observed in microscope (Olympus). The leaves of the same part of the sample tested were selected, net photosynthetic rate (*P*n), stomatal conductance (*G*s), intercellular CO_2_ (*C*i) and transpiration rate (*T*r) were measured on the third leaf from top to bottom using LI-6400 photosynthetic apparatus. The light intensity of the instrument was set to 1000 μmol m^−2^s^−1^, the gas flow rate was set to 500 μmol s^−1^ and the CO_2_ concentration was set to 400 μmol mol^−1^. The chlorophyll content of tobacco was measured by SPAD chlorophyll meter (Tokyo, Japan). The method of O^2−^ free radical scavenging ability determination refers to [32].

NBT and DAB staining kit was purchased from Tiangen Biotech co., LTD (Beijing, China). The protocol follows the instructions of the kit.

Hydrogen peroxide (H_2_O_2_) kit was purchased from Grace Biotechnology co., LTD (Suzhou, China). The UV absorption value was measured at 405 nm according to the specific experimental operation method, and the H_2_O_2_ content was calculated according to the formula in the operation instructions. The content of malondialdehyde (MDA) in leaves was determined using the thiobarbituric acid (TBA) method [33].

### 4.7. Screening and Validation of LpPsbP-Interacting Proteins

The recombinant plasmid pGBKT7-LpPsbP was obtained by connecting the *LpPsbP* gene and pGBKT7 vector with BamH1 and Sal1 restriction site. pGBKT7-LpPsbP plasmid was transferred into Y2H with the screening of cDNA library. Additionally, the blue colonies were selected from SD-Trp-Leu-His plus X-α-gal medium. DNA was used as the template, while *T7* and *3′-AD* as upstream and downstream primers for PCR identification and the product of PCR was sequenced. 

pGBKT7-LpPsbP and interacting protein-pGAD17 were co-transformed into Y2H Gold to examine their interactions. The colonies were cultured on SD-Trp-Leu-His plus X-α-gal medium. pGBKT7-53 plus pGADT7-T was used as positive control, and pGBKT7-lam plus pGADT7-T was used as negative control.

*LpFoxO* gene was cloned by PCR using *L. pumilum* cDNA as template (*LpFoxO-F* and *LpFoxO-R*, shown in Table 1). The *LpFoxO* and pGAD17 vectors were ligated through BamH1 and Xho1 restriction site. pGBKT7-LpPsbP and pGADT7-LpFoxO were co-transformed into Y2HGold. Then, the co-transformed bacterial liquid was placed in SD-Trp-Leu-His plus X-α-gal medium. Co-transformed strains of pGBKT7 and pGADT7, co-transformed strains of pGADT7 and pGBKT7-LpPsbP and co-transformed strains of pGBKT7 and pGADT7-LpFoxO were used as control groups.

The *LpPsbP* gene was constructed into the pCAMBIA1300-NLuc vector (primers: *LpPsbP-SacI-F* and *LpPsbP-SalI-R*, shown in Table 1) and the *FoxO* gene was cloned into pCAMBIA1300-CLuc vector (primers: *FoxO-BamHI-F* and *FoxO-SalI-R*, shown in Table 1). The constructed vector plasmids were transformed into *Agrobacterium* GV1301, and then injected into tobacco leaves. The leaves are exposed to the dark for two days before being injected with D-fluorescein potassium salt and then left in the dark for five minutes. The photos were taken using the Tanon-5200 Chemiluminescent Imaging System (Shanghai, China).

### 4.8. Cloning of the LpPsbP Promoter

The sequence of *LpPsbP* promoter was cloned using Takara chromosome walking kit (Tokyo, Japan) and analyzed using PlantCARE (http://bioinformatics.psb.ugent.be/webtools/plantcare/html/ (accessed on 7 January 2023)). Promoter cloning primers are *LpPsbP SP1*, *LpPsbP SP2*, and *LpPsbP SP3* (shown in Table 1).

## 5. Conclusions

In this study, the *LpPsbP* gene was cloned from the bulbs of *L. pumilum*, and the following conclusions were drawn: 1. The PsbP protein is closely related to the PsbP of *A. officinalis*, *F. agrestis* and other plants. LpPsbP is highly conserved in domain DcrB, which belongs to the DcrB superfamily. 2. The maximal expression of the protein is obtained under the induction with 1 mM IPTG for 1 h. The pGEX-LpPsbP purified protein was obtained. The tolerance of bacteria solution including LpPsbP-GST fusion protein is higher than that only including the GST protein. 3. Transgenic tobacco plants are subjected to NaCl, Na_2_CO_3_, NaHCO_3_ and H_2_O_2_ stress. Compared with wild-type plants, seed germination, seedling growth, phenotypes of mature tobacco under stress treatment, leaf stomatal size, O^2−^ and H_2_O_2_ staining in leaves were observed. The physiological parameters such as net photosynthetic rate (*P*n), stomatal conductance (*G*s), intercellular CO_2_ (*C*i) and transpiration rate (*T*r), as well as chlorophyll content, superoxide anion (O^2−^) content, H_2_O_2_ content and malondialdehyde (MDA) content of wild-type and transgenic lines under salt stress were measured. The presence of the *LpPsbP* gene can alleviate the reduction in gas exchange function in leaves induced by salt stress, reduce the accumulation of reactive oxygen species (ROS) and alleviate plant damage. 4. A total of 18 interacting fragments are screened out. The interaction between pGBKT7-LpPsbP and pGADT7-LpFoxO is verified by yeast two-hybrid. Moreover, the results of luciferase complementation assay further showed that LpPsbP and FoxO can interact with each other. 5. There are many cis-acting elements involved in the response of abiotic stresses, light and hormones in the promoter of *LpPsbP*, which ensures that this promoter contributes significantly to many biological metabolic pathways.

**Figure 8:**
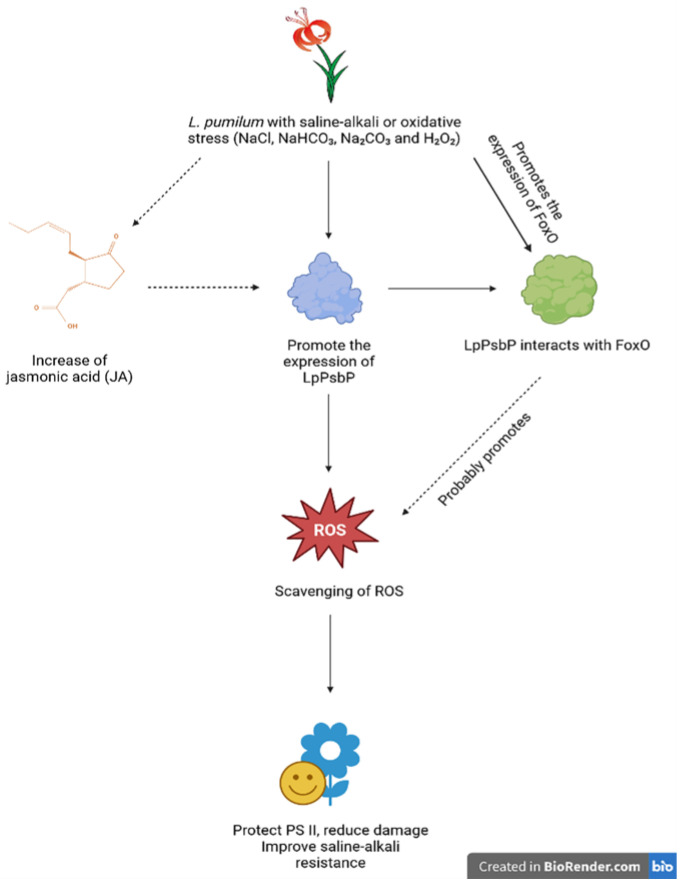
Concluded mechanism of how LpPsbP improves Saline-Alkali resistance in *L. pumilum*. After Saline-Alkaline stress and oxidative stress (caused by NaCl, NaHCO_3_, Na_2_CO_3_ and H_2_O_2_), *L. pumilum* creates ROS at a variety of locations, including chloroplasts. The sub-site of ROS generation in chloroplasts is mostly at the center of the oxygen-releasing complex. When encountering these stresses, the expression of LpPsbP is promoted. Then, LpPsbP will scavenge ROS, which means it will finally safeguard the PS II of *L. pumilum*, lessen damage to it, and therefore increase the ability of plants to tolerate stress. Moreover, we have made two additional speculations: 1. Following stress, *L. pumilum* generates more JA, which directly promotes the expression of the *PsbP* gene. 2. First comes with the fact that the *FoxO* gene will express itself more when ROS are created, which means that *L. pumilum* encounters these two kinds of stress. Then, because these two ROS scavenging proteins (LpPsbP and FoxO) can interact with each other, it will promote the scavenging of ROS, leading to a better protection and resistance. This figure is made using BioRender website (https://biorender.com/), dashed lines in this figure means these are speculations instead of definitive knowledge.

Considering the above, we eventually conclude that LpPsbP may improve the Saline-Alkali resistance of *L. pumilum* mainly through the molecular pathway of scavenging ROS: *L. pumilum* produces ROS at sites including chloroplasts after Saline-Alkali stress or oxidative stress (such as NaCl, NaHCO_3_, Na_2_CO_3_ and H_2_O_2_), and the sub-site of ROS production in chloroplasts is mainly at the center of the oxygen releasing complex. When *L. pumilum* encounters Saline-Alkali stress or oxidative stress, the expression level of LpPsbP will be increased. Then, by directly scavenging ROS, LpPsbP will protect the PS II of *L. pumilum*, reduce its damage and thus improve the Saline-Alkali resistance of the plant. Moreover, we speculate that Saline-Alkali stress or oxidative stress will also promote the production of jasmonic acid (JA), leading to another promotion pathway of LpPsbP expression. Additionally, another speculation we have is that because LpPsbP can interact with FoxO and both of them can scavenge ROS alone, this interacting complex will probably promote the scavenging of ROS, leading to a better protection and resistance (Figure 8). 

## Figures and Tables

**Table 1:** Primers used in this study.

Primer Name	Primer Sequence
*LpPsbP-F*	ATGGCCTCATCTGCATGC
*LpPsbP-R*	TCATGCAACATTGAAGGA
*LpPsbP qPCR-F*	CACCAACCATCAAGCCCTCT
*LpPsbP qPCR-R*	CCTGACCTGGGAACTCAACC
*LpPsbP-BamH1-F*	GGATCCATGGCCTCATCTGCAT
*LpPsbP-Xho1-R*	CTCGAGTCATGCAACATTGAAGGA
*LpPsbP-Sal1-R*	GTCGACTCATGCAACATTGAAGGA
*LpFoxO-F*	ATGTCGATTAGCCGGGCA
*LpFoxO-R*	TCATCTCCCAGCACAGTT
*pGEX-LpPsbP-BamH1-F*	GGATCCATGGCCTCATCTGCAT
*LpPsbP SP1*	ATGGCAGCAGAGCCAATGAGGA
*LpPsbP SP2*	TTCTGAGCCCGACAGATGAGCT
*LpPsbP SP3*	ACATGGTGGAGGAGGAAGCATG
*T7*	TAATACGACTCACTATAGGGC
*3′-AD*	AGATGGTCACGATGCACAG
*LpPsbP-SacI-F*	GAGCTCATGGCATCGACAGC
*LpPsbP-SalI-R*	GTCGACAGCAACATTGAAGG
*FoxO-BamHI-F*	GGATCCATGTCGATTAGCCG
*FoxO-SalI-R*	GTCGACTCATCTCCCAGCAC

**Table 2:** The proteins coded by 5 sequences related to Saline-Alkali resistance.

Protein Name	Functions	Reference
Winged-helix DNA-binding transcription factor family protein	Modulate DNA damage response through the regulation of *SOG1* and *ATR* transcription level.	[17]
Pollen Ole e 1 allergen and extensin family protein	Response to salt stress by maintain the steady state of endoplasmic reticulum.	[18]
Phosphatidyl glycerol phosphate synthase 2	Stabilizing photosynthetic membrane by producing phosphatidyl glycerol phosphate in order to maintain the stability of plant under Saline-Alkali stress.	[19]
Putative lysine decarboxylase family protein	Play an important role in direct activation pathway of cytokinesis.	[20]
ARF-GAP domain 13	Release dormancy of plant stem by affecting the transport of vesicle.	[21]

**Table 3:** Analysis of cis-acting elements in the promoter sequence of *LpPsbP* gene.

Cis-Element	Motif (5′-3′)	Function	Frequency
ABRE	ACGTG	Cis-acting element involved in the abscisic acid responsiveness.	1
ARE	AAACCA	Cis-acting regulatory element essential for the anaerobic induction.	2
CGTCA-motif	CGTCA	Cis-acting regulatory element involved in the MeJA-responsiveness.	1
GATA-motif	GATAGGG	Part of a light responsive element.	2
GC-motif	CCCCCG	Enhancer-like element involved in anoxic specific inducibility.	2
GT1-motif	GGTTAAT	Light responsive element.	1
P-box	CCTTTTG	Gibberellin-responsive element.	1
TGACG-motif	TGACG	Cis-acting regulatory element involved in the MeJA-responsiveness.	1
Circadian	CAAAGATATC	Cis-acting regulatory element involved in circadian control.	1

## Data Availability

Not applicable.

## Abbreviations

ROS	Reactive oxygen species
JA	Jasmonic acid
PS II	Photosystem II
PsbP	PS II subunit P
IPTG	Isopropyl-beta-D-thiogalactopyranoside
GST	Glutathione S-transferase
OD	Optical density
NBT	Nitro-blue tetrazolium
DAB	Diaminobenzidine
MDA	Malondialdehyde
TBA	Thiobarbituric acid
